# The complete mitochondrial genome sequence of *Photopectoralis bindus* (Perciformes: Leiognathidae)

**DOI:** 10.1080/23802359.2017.1422404

**Published:** 2018-01-03

**Authors:** Wei Shi, Baosheng Wu, Hui Yu

**Affiliations:** a College of Life Science, Foshan University, Foshan, Guangdong, PR China;; b CAS Key Laboratory of Tropical Marine Bio-Resources and Ecology, South China Sea Institute of Oceanology, Chinese Academy of Sciences, Guangzhou, PR China

**Keywords:** Complete mitochondrial genome, *Photopectoralis bindus*, phylogenetic relationship

## Abstract

The complete mitochondrial genome of *Photopectoralis bindus* was sequenced by high throughput sequencing method in this study. Length of this genome is 16,517 bp, containing 13 protein-coding genes, 22 tRNA genes, two rRNA genes and one large non-coding region. *ND6* and eight tRNA genes are encoded by L-strand, and others are encoded by H-strand, which is similar to those in most vertebrates. The nucleotide composition of the whole mitogenome is 29.9% A, 30.1% C, 15.1% G, and 25.0% T, with a slight bias of A + T content (54.9%). Phylogenetic tree based on the first and second codon sequences of 12 protein coding genes (except *ND6*) shows that the clade of *P. bindus* is closely clustered with that of *Gazza minuta*, and families Rachycentridae and Carangidae have the closest relationship to Leiognathidae.

*Photopectoralis bindus* is a common coastal water fish, whose body greatly compressed and slimy, head usually naked, and upper surface with bony ridges. It mainly inhabits in the Indian – western Pacific Ocean, few distributed along the Mediterranean coast. In this study, *P*. *bindus* was caught in Naozhou island in Zhanjiang (geographic coordinate: N 20°53′20.11″, E 112°28′46.2″). The specimen was preserved in ethanol and registered to the Marine Biodiversity Collection of South China Sea, Chinese Academy of Sciences, under the voucher number SCF20171022015. This study first determined the complete mitochondrial genome sequence of *P. bindus* and analyzed its phylogenetic relationships within order Perciformes.

The complete mitochondrial genome of *P. bindus* has 16,795 bp in length (GenBank accession no. MG677547), including 13 protein-coding genes, two ribosomal RNA (rRNA) genes, 22 transfer RNA (tRNA) genes and one control region (D-Loop). Genes encoding on the genome are similar to other vertebrates (Yu and Kwak 2015). The 22 tRNA genes were interspersed between rRNAs and protein-coding genes, with various sizes ranging from 67 bp (*tRNA-Ser*) to 74 bp (*tRNA-Leu, Thr and Lys*). The OL (origin of replication on the light-strand) was found in the cluster of five tRNA genes (*WANCY* region) between *tRNA-Asn* and *tRNA-Cys*. And the D-Loop (control region) was 854 bp in length, between *tRNA-Pro* and *tRNA-Phe*. The 12 protein-coding genes used the initiation codon ATG and the rest one (*COI*) started with GTG, which is as same as *Brachirus orientalis* (Shi et al. 2016). Moreover, nine protein-coding genes used TAA as the stop codon, The *ND3* and *ND6* ended with TAG, *ND4* ended with TA, while *CO II* ended with just a single base T, which is different from the common stop codon with three bases. And same situation were also found in many other fishes, such as *Liobagrus styani* (Huang et al. 2017), *Zebrias quagga* (Li et al. 2016). Except the *ND6* and eight tRNA genes encoded on the L-strand, most of these genes are encoded by the H-strand, which is similar to those of other vertebrates. The nucleotide composition of the whole mitogenome of *P. bindus* is T: 25.0%, C: 30.1%, A: 29.9% and G: 15.1%.

The phylogenetic relationship of *P. bindus* was deduced by its comparison with other nine closely related species, whose complete mitochondrial genes are available on GenBank. The Neighbor-Joining evolutionary tree (NJ tree) was constructed by MEGA 7 (Kumar et al. 2016) based on first and second codon sequences of 12 protein coding genes (except *ND6*), with *Lutjanus kasmira* of Lutjanidae family as an outgroup. In the NJ tree, *P. bindus* clustered with *Gazza minuta* with a strong support, *Rachycentron canadum*, *Echeneis naucrates*, *Trachinotus ovatus* and *Alectis ciliaris* formed a clade as a sister lineage to clade of *P. bindus* and *G. minuta* (Figure 1). These results show that families Rachycentridae, Echeneidae and Carangidae have closer phylogenetic relationship to Leiognathidae.

**Figure 1.**
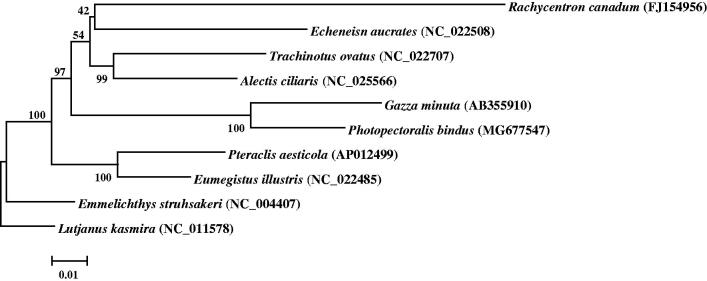
Neighbor-Joining tree was constructed based on first and second codon sequences of 12 protein coding genes of 10 species.
